# The reciprocal regulation loop of Notch2 pathway and miR-23b in controlling gastric carcinogenesis

**DOI:** 10.18632/oncotarget.4000

**Published:** 2015-05-18

**Authors:** Tzu-Ting Huang, Yueh-Hsin Ping, An-Ming Wang, Chia-Chi Ke, Wen-Liang Fang, Kuo-Hung Huang, Hsin-Chen Lee, Chin-Wen Chi, Tien-Shun Yeh

**Affiliations:** ^1^ Department and Institute of Pharmacology, School of Medicine, National Yang-Ming University, Taipei, Taiwan; ^2^ Department of Anatomy and Cell Biology, School of Medicine, National Yang-Ming University, Taipei, Taiwan; ^3^ Institute of Clinical Medicine, School of Medicine, National Yang-Ming University, Taipei, Taiwan; ^4^ Department of Surgery, Taipei Veterans General Hospital, Taipei, Taiwan; ^5^ Department of Medical Research, Taipei Veterans General Hospital, Taipei, Taiwan; ^6^ Institute of Biochemistry and Molecular Biology, National Yang-Ming University, Taipei, Taiwan; ^7^ Genome Research Center, National Yang-Ming University, Taipei, Taiwan; ^8^ Graduate Institute of Medical Sciences, College of Medicine, Taipei Medical University, Taipei, Taiwan

**Keywords:** notch2 receptor, miR-23b, Ets1, E2F1, gastric carcinogenesis

## Abstract

Gastric carcinoma is one of the most common malignancies and the third highest cause of global cancer-related death. Notch2 receptor intracellular domain (N2IC), the activated form of Notch2 receptor, enhances gastric carcinogenesis. MicroRNAs (miRNAs) act as either oncogenes or tumor suppressors in tumorigenesis and cross-talk with Notch pathways. Herein, microRNA-23b (miR-23b) was identified as a Notch2 receptor-related miRNA and its role in gastric carcinogenesis was investigated. Levels of miR-23b in stomach adenocarcinoma samples were down-regulated, whereas those of Notch2 receptor, v-ets erythroblastosis virus E26 oncogene homolog 1 (Ets1), and E2F1 transcripts were up-regulated. Results also showed that N2IC down-regulated miR-23b expression in gastric cancer cells through up-regulating E2F1. The miR-23b inhibited gastric tumorigenesis including growth, viability, epithelial-mesenchymal transition, and abilities of colony formation, migration, invasion, and tumorsphere formation. Mechanistically, miR-23b suppressed tumor progression and pluripotency gene expression and affected tumorsphere ultra-structure in gastric cancer cells *via* targeting Notch2 receptor or Ets1. Furthermore, miR-23b diminished the xenografted tumor growth and lung metastasis of SC-M1 gastric cancer cells through Notch2 pathway. Our results suggest that Notch2 pathway and miR-23b interplay in a reciprocal regulation loop in gastric cancer cells and this axis plays an important role in gastric carcinogenesis.

## INTRODUCTION

Gastric carcinoma is the third leading cause of global cancer mortality [1]. The regulatory mechanisms modulating aggressiveness of gastric cancer are incompletely elucidated. There are four Notch receptors (Notch1–4) in mammals [2, 3]. Notch receptors are activated after cleavage induced by ligand-binding to release their Notch receptor intracellular domains, the activated forms of Notch receptors. Notch receptors act as oncogenes or tumor suppressors in controlling tumorigenesis [2, 3]. In gastric cancer cells, Notch1 and Notch2 receptor intracellular domains (N1IC and N2IC, respectively) enhance carcinogenesis through up-regulating cyclooxygenase-2 [4, 5]. Furthermore, Notch2 receptor mRNA is up-regulated in the population of cancer stem cells [6].

MicroRNAs (miRNAs) are small non-coding RNAs that bind to the 3′-untranslated regions (3′-UTRs) of target mRNAs [7, 8]. A growing body of evidence indicates that miRNAs function as either oncogenes or tumor suppressors in the development and progression of human malignancies [8, 9]. Notch pathways regulate expressions of a number of miRNAs, while several miRNAs modulate levels of the components in Notch pathways [10, 11]. For example, Notch1 pathway interplays with several miRNAs in cancer cells [10, 12]. The Notch3-regulated miR-223 represses the tumor suppressor FBXW7 in T-cell acute lymphoblastic leukemia [13]. Additionally, miR-34 family down-regulates Notch1 and Notch2 levels in gastric cancer cells [14] and also inhibits self-renewal of pancreatic cancer stem cells *via* directly modulating Notch1 and Notch2 receptors [15]. miR-107 suppresses growth and metastasis of brain tumor cells through down-regulating Notch2 receptor [16, 17]. Notch2 pathway/miR-205 reciprocal regulation loop regulates mammary stem cell fate [18]. These results showed a significant cross-talk between Notch pathways and miRNAs in carcinogenesis. In the present study, we sought to search for the Notch2 receptor-related miRNAs which are involved in controlling tumor development and progression of gastric cancer cells. miR-23b was identified as a Notch2 receptor-related miRNA and its role in controlling gastric tumorigenesis was further investigated.

## RESULTS

### Levels of miR-23b are down-regulated in stomach adenocarcinoma samples, whereas transcripts of Notch2 receptor, Ets1, and E2F1 are up-regulated

To identify the Notch2 receptor-related miRNAs in gastric cancer cells, miRNA quantitative real-time PCR analysis was performed in N2IC-expressing human stomach adenocarcinoma SC-M1 cells (SC-M1/myc-N2IC-His cells) and control cells. We found that miR-23b was the most potent Notch2 pathway-suppressing miRNA (data not shown). To survey whether any significant difference of levels of miR-23b and Notch2 receptor mRNA exists in stomach adenocarcinoma specimens compared with those of normal tissues, data from The Cancer Genome Atlas (TCGA) were analyzed. Furthermore, mRNAs of the known cellular factors regulating gastric carcinogenesis were also examined including E2F1 [19, 20] and Ets1 [21].

**Figure 1:**
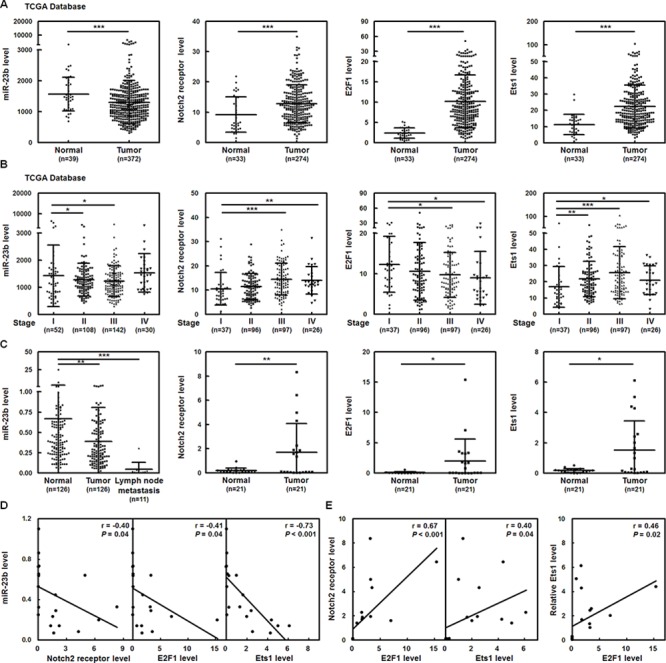
Levels of miR-23b are down-regulated in stomach adenocarcinoma samples, whereas transcripts of Notch2 receptor, Ets1, and E2F1 are up-regulated **A.** Level 3 data of mRNA and miRNA expressions from stomach adenocarcinoma samples and normal tissue samples were downloaded from the TCGA and Broad GDAC Firehose data portal. The mRNA RPKM (Reads per Kilobase of exon model per Million) and microRNA reads per million mappable reads of all samples were selected and analyzed for comparing abundances by GraphPad Prism 5 software. The transcript levels of miR-23b, Notch2 receptor, E2F1, and Ets1 in stomach adenocarcinoma samples (miR-23b, *n* = 372; Notch2, E2F1, and Ets1, *n* = 274) and normal tissue samples (miR-23b, *n* = 39; Notch2, E2F1, and Ets1, *n* = 33) were measured by RNA sequencing in TCGA data. ***P < 0.001. **B.** The transcript levels of miR-23b, Notch2 receptor, E2F1, and Ets1 in stomach adenocarcinoma samples were downloaded and then divided according to the stage classification. Transcript levels of miR-23b, Notch2, E2F1, and Ets1 in the gastric cancer tissues of stages II to IV were compared with those of early stage I. *P < 0.05; **P < 0.01; ***P < 0.001. **C.** Tumor, the adjacent non-tumor tissue, and lymph-node tumor sample pairs from gastric cancer patients (miR-23b in tumor and the adjacent non-tumor tissue, *n* = 126; miR-23b in lymph-node tumor samples, *n* = 11; Notch2, Ets1, and E2F1, *n* = 21) were examined using quantitative real-time PCR analysis. Levels of miR-23b and Notch2, E2F1, and Ets1 mRNAs in the gastric cancer tissues or lymph-node tumor samples were compared with those of the corresponding adjacent normal tissues. *P < 0.05; **P < 0.01; ***P < 0.001. Data are shown as mean ± standard deviation. **D-E.** The correlation between miR-23b and Notch2 receptor, E2F1, or Ets1 mRNA expressions D and among Notch2 receptor, E2F1, and Ets1 mRNA expressions E in the tumors from 21 gastric cancer patients was analyzed by Pearson correlation analysis.

Results showed that expressions of miR-23b were significantly down-regulated in numerous stomach adenocarcinoma samples compared with normal counterparts, whereas those of Notch2 receptor, E2F1, and Ets1 mRNAs were up-regulated (Figure 1A). According to stage classification, mRNA expressions of Notch2 receptor and Ets1 but not E2F1 were increased in stomach adenocarcinoma specimens from patients with gastric cancer advanced stages II–IV, compared with early stage I, whereas levels of miR-23b were decreased (Figure 1B). The miR-23b-27b-24-1 cluster is composed of miR-23b, miR-27b, and miR-24-1. We also found that levels of miR-27b but not miR-24-1 with rare expression were suppressed in gastric cancer samples (Supplementary Figure S1A) and inhibited in those specimens from patients with advanced stages (Supplementary Figure S1B).

Additionally, the quantitative real-time PCR analysis was employed on gastric cancer samples and the corresponding adjacent normal tissues of gastric cancer patients to examine their clinical relevance. Levels of miR-23b were lower in stomach adenocarcinoma specimens than in their corresponding normal tissue counterparts, but those of Notch2 receptor, E2F1, and Ets1 mRNAs were higher (Figure 1C). Notably, the miR-23b expressions were dramatically down-regulated in lymph-node tumor samples from 11 gastric cancer patients with lymph node metastasis. The relationship between levels of miR-23b and Notch2 receptor, E2F1, and Ets1 mRNAs in the tumor samples from 21 gastric cancer patients was analyzed using the Pearson correlation analysis. The relative miR-23b levels were inversely proportional to the expressions of Notch2 receptor, E2F1, and Ets1 mRNAs in stomach adenocarcinoma specimens (Figure 1D). Moreover, the relative levels of Notch2 receptor, E2F1 and Ets1 mRNAs in these samples were positively correlated with those of each other (Figure 1E).

### N2IC down-regulates miR-23b expression in gastric cancer cells

**Figure 2:**
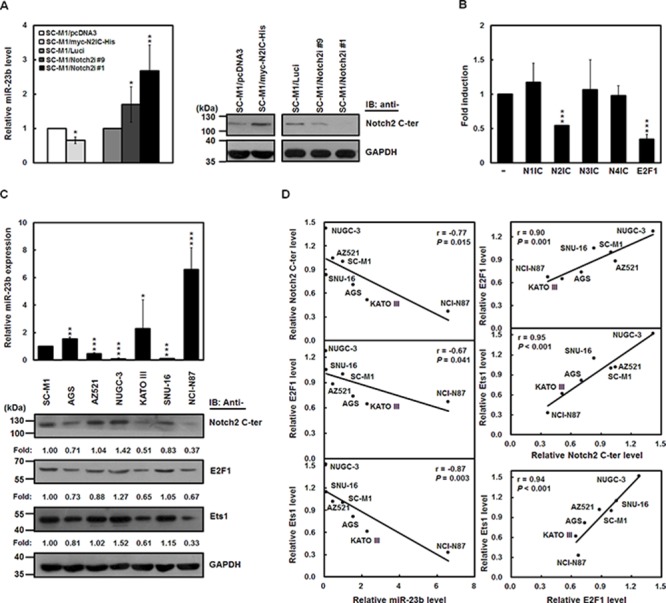
N2IC down-regulates miR-23b expression in gastric cancer cells **A.** The relative levels of miR-23b in N2IC-expressing SC-M1/myc-N2IC-His cells, Notch2 receptor-knocked down SC-M1/Notch2i cells (#1 and #9), and their control cells (SC-M1/pcDNA3 cells and SC-M1/Luci cells, respectively) were determined using miRNA quantitative real-time PCR (*left*). The levels of miR-23b in control cells were set to unity. Whole-cell extracts of these cells were analyzed by Western blot analysis using anti-Notch2 C-terminal (C-ter) and anti-GAPDH antibodies (*right*). *P < 0.05; **P < 0.01. **B.** Reporter plasmid containing miR-23b-27b-24-1 promoter was co-transfected with expression constructs of Notch1 receptor (N1IC), Notch2 receptor (N2IC), Notch3 receptor (N3IC), and Notch4 receptor (N4IC) intracellular domains as well as E2F1 or control vector pcDNA3.1-myc-His (−) into SC-M1 cells for 48 hours for reporter gene assay. ***P < 0.001. **C.** The relative levels of endogenous miR-23b in human gastric cancer cells were determined using miRNA quantitative real-time PCR (*upper*) including SC-M1, AGS, AZ521, NUGC-3, KATO III, SNU-16, and NCI-N87 cells. Level of miR-23b in SC-M1 cells was set to unity. *P < 0.05; **P < 0.01; ***P < 0.001. Whole-cell extracts of these cells were also analyzed by Western blot analysis using anti-Notch2 C-terminal (C-ter), anti-E2F1, anti-Ets1, and anti-GAPDH antibodies (*lower*). The intensities were quantified using Multi-Gouge V3.0 and then normalized to the internal control GAPDH. The results were calibrated to levels of the detected proteins in SC-M1 cells. Data are shown as mean ± standard deviation. **D.** As described above, the correlation among miR-23b, N2IC, E2F1, and Ets1 transcript expressions in seven gastric cancer cells was analyzed by Pearson correlation analysis.

To confirm the N2IC-mediated miR-23b suppression, miR-23b levels were examined in N2IC-expressing SC-M1/myc-N2IC-His cells, and Notch2 receptor-knocked down SC-M1/Notch2i cells by analysis of miRNA quantitative real-time PCR (Figure 2A). Results showed that miR-23b expression was decreased by N2IC overexpression, whereas increased by Notch2 receptor knockdown.

To evaluate whether four Notch pathways suppress miR-23b expression by inhibiting its promoter activity, reporter plasmid containing miR-23b-27b-24-1 promoter was co-transfected with constructs of Notch receptor intracellular domains into SC-M1 cells for reporter gene assay (Figure 2B). Only the exogenous N2IC significantly inhibited the activity of miR-23b-27b-24-1 promoter among four Notch receptor intracellular domains.

Using search engines PROMO and TFSEARCH to identify transcription factor-binding sites [22, 23], there are putative E2F1-binding sites in human miR-23b-27b-24-1 promoter (Supplementary Figure S2A). As demonstrated previously [24], miR-23b-27b-24-1 promoter activity was suppressed by c-Myc, a downstream target of Notch pathway [25–28]. Because c-Myc and E2F1 activate each other's transcription [29, 30], we checked whether Notch2 pathway suppresses miR-23b expression through E2F1 in gastric cancer cells. Data showed that E2F1 and c-Myc expressions were enhanced in N2IC-expressing SC-M1/myc-N2IC-His cells, whereas decreased in Notch2 receptor-knocked down SC-M1/Notch2i cells by quantitative real-time PCR and Western blot analyses (Supplementary Figure S2B). Furthermore, miR-23b levels were increased after transfection with siRNA vectors against E2F1 into SC-M1 cells (Supplementary Figure S2C). The N2IC-mediated miR-23b suppression was relieved by E2F1 knockdown when SC-M1 cells were co-transfected with N2IC-expressing construct and siRNA vectors against E2F1.

The relative levels of endogenous miR-23b in seven human gastric cancer cells were also determined using miRNA quantitative real-time PCR (Figure 2C, *upper*). miR-23b was expressed in SC-M1, AGS, KATO III, and NCI-N87 cells but hardly detected in AZ521, NUGC-3, and SNU-16 cells. Results of Western blot analysis showed that N2IC, E2F1, and Ets1 were differentially expressed in these cells (Figure 2C, *lower*). Using the Pearson correlation analysis, the relative miR-23b levels were inversely proportional to N2IC, E2F1, and Ets1 expressions in these cells (Figure 2D, *left*), whereas the relative levels of N2IC, E2F1, and Ets1 were positively correlated with those of each other (Figure 2D, *right*).

### miR-23b inhibits growth and progression of gastric cancer cells

**Figure 3:**
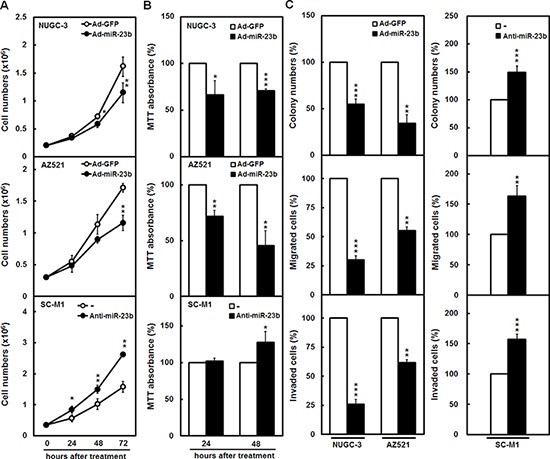
miR-23b inhibits growth and progression of gastric cancer cells **A-C.** NUGC-3 and AZ521 cells were infected with adenoviruses expressing miR-23b (Ad-miR-23b) or GFP (Ad-GFP) and SC-M1 cells were transfected with 100 nM antagomir-23b (anti-miR-23b) or scrambled control (−). The infected NUGC-3 (*upper*) as well as AZ521 cells (*middle*) and transfected SC-M1 cells (*lower*) were seeded and then counted by trypan blue exclusion method A or incubated to analyze cell viability by MTT assay B at the time indicated. The infected NUGC-3 as well as AZ521 cells (*left*) and transfected SC-M1 cells (*right*) were seeded for colony formation (*upper*), migration (*middle*), and invasion (*lower*) assays C Means of at least three independent experiments performed in triplicate are shown. *P < 0.05; **P < 0.01; ***P < 0.001. Data are shown as mean ± standard deviation.

To study the roles of miR-23b in gastric tumorigenesis, miR-23b-expressing adenoviral system was established. As revealed by miRNA quantitative real-time PCR analysis (Supplementary Figure S3), miR-23b levels were increased in NUGC-3 as well as AZ521 cells scarcely expressing miR-23b and SC-M1 cells expressing miR-23b after infection with miR-23b-expressing adenoviruses as compared with those infected with green fluorescent protein (GFP)-expressing adenoviruses. Alternatively, the antagomir-23b reagent, chemically modified antisense RNA oligonucleotides, was used to inhibit the function of miR-23b and to address the role of endogenous miR-23b in gastric carcinogenesis. Transient transfection with 50 or 100 nM of antagomir-23b significantly knocked down miR-23b expression in SC-M1 cells (Supplementary Figure S4). To explore whether miR-23b regulates growth of gastric cancer cells, trypan blue exclusion method and MTT assay were employed. After infection with miR-23b-expressing adenoviruses, the cumulative numbers of NUGC-3 and AZ521 cells were reduced (Figure 3A). The cumulative numbers of SC-M1 cells were increased after transfection with antagomir-23b (Figure 3A). Results of MTT assay showed that the viabilities were inhibited by miR-23b overexpression in NUGC-3 and AZ521 cells, but increased by miR-23b knockdown in SC-M1 cells (Figure 3B).

To evaluate whether miR-23b regulates gastric cancer progression, NUGC-3 and AZ521 cells were infected with miR-23b-expressing adenoviruses for overexpression and SC-M1 cells were transfected with antagomir-23b for knockdown and used for the subsequent colony formation, migration, and invasion assays. Progression abilities were diminished after miR-23b overexpression in NUGC-3 and AZ521 cells (Figure 3C, *left*), whereas augmented after miR-23b knockdown in SC-M1 cells (Figure 3C, *right*).

### miR-23b suppresses epithelial-mesenchymal transition (EMT) and sphere formation ability of gastric cancer cells

**Figure 4:**
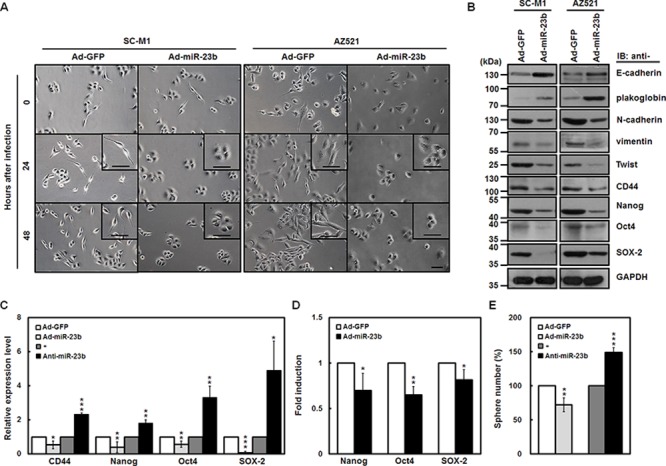
miR-23b suppresses EMT and sphere formation ability of gastric cancer cells **A.** SC-M1 (*left*) and AZ521 (*right*) cells were infected with adenoviruses expressing miR-23b (Ad-miR-23b) or GFP (Ad-GFP) and subsequently seeded onto 6-well plates for 24 or 48 hours for morphological examination. Bar, 50 μm. **B.** Whole-cell extracts of the infected SC-M1 (*left*) and AZ521 (*right*) cells were prepared for Western blot analysis using anti-E-cadherin, anti-plakoglobin, anti-N-cadherin, anti-vimentin, anti-Twist, anti-CD44, anti-Nanog, anti-Oct4, anti-SOX-2, and anti-GAPDH antibodies. **C.** After infection with adenoviruses expressing miR-23b or GFP or transfection with 100 nM antagomir-23b (anti-miR-23b) or scrambled control (−) into SC-M1 cells, the transcript levels of CD44, Nanog, Oct4, and SOX-2 were measured by quantitative real-time PCR and then normalized to GAPDH. *P < 0.05; **P < 0.01; ***P < 0.001. **D.** After transfection with reporter plasmids Nanog-Luc (Nanog), Oct4-Luc (Oct4), or SOX-2-Luc (SOX-2) for 24 hours, SC-M1 cells were infected with adenoviruses expressing miR-23b or GFP for 24 hours for reporter gene assay. *P < 0.05; **P < 0.01. **E.** After infection with adenoviruses expressing miR-23b or GFP or transfection with 100 nM antagomir-23b or scrambled control into SC-M1 cells for 48 hours, the treated cells were seeded and then incubated for 9 days for tumorsphere formation assay. **P < 0.01; ***P < 0.001. Data are shown as mean ± standard deviation.

Next, we sought to check whether miR-23b regulates EMT of gastric cancer cells. SC-M1 and AZ521 cells with adherent growth property were infected with miR-23b-expressing adenoviruses, and the infected cells had a slightly packed morphology compared with those infected with GFP-expressing adenoviruses (Figure 4A). Expressions of epithelial markers E-cadherin and plakoglobin were enhanced along with the decreased levels of mesenchymal markers N-cadherin, vimentin, and Twist by miR-23b overexpression in SC-M1 and AZ521 cells using Western blot analysis (Figure 4B). Interestingly, the blockade of pluripotency genes including CD44, Nanog, Oct4, and SOX-2 was also exerted by miR-23b overexpression (Figure 4B). The mRNA levels of these pluripotency genes in SC-M1 cells were inhibited by miR-23b overexpression, but enhanced by miR-23b knockdown (Figure 4C). Data of reporter gene assay showed that the activities of Nanog, Oct4, and SOX-2 promoters were inhibited by miR-23b overexpression (Figure 4D). Furthermore, tumorsphere formation ability of SC-M1 cells was repressed after miR-23b overexpression, whereas enhanced after miR-23b knockdown (Figure 4E).

### miR-23b attenuates progression of gastric cancer cells through Notch2 pathway or Ets1

**Figure 5:**
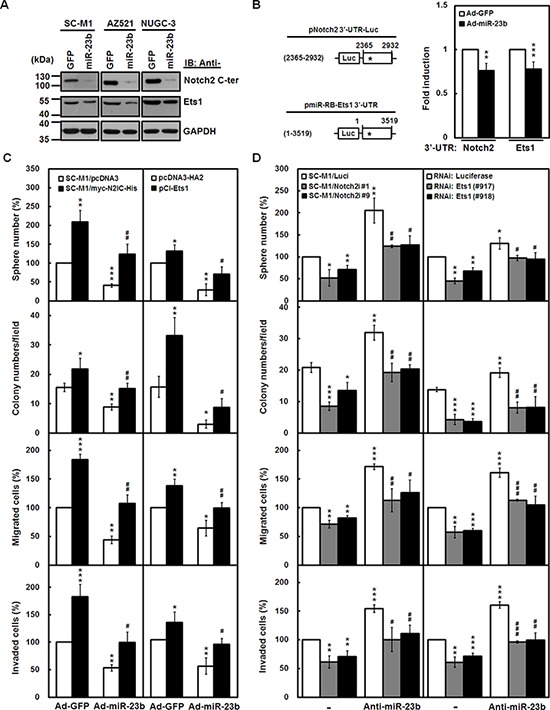
miR-23b attenuates progression of gastric cancer cells through Notch2 pathway or Ets1 **A.** SC-M1, AZ521, and NUGC-3 cells were infected with adenoviruses expressing miR-23b (Ad-miR-23b) or GFP (Ad-GFP) for 48 hours. Whole-cell extracts of the infected cells were prepared for Western blot analysis using anti-Notch2 C-terminal (C-ter), anti-Ets1, and anti-GAPDH antibodies. **B.** Schematic representation of luciferase reporter plasmids pNotch2 3′-UTR-Luc (2365–2932) and pmiR-RB-Ets1 3′-UTR containing DNA fragments of human Notch2 receptor (nucleotide 2365 to 2932) and Ets1 (nucleotide 1–3519) 3′-UTRs, respectively (*left*). Star indicates position of the putative miR-23b-binding site in reporter plasmids. After transfection with reporter plasmids pNotch2 3′-UTR-Luc (2365-2932) and pmiR-RB-Ets1 3′-UTR for 24 hours, SC-M1 cells were infected with adenoviruses expressing miR-23b or GFP for 24 hours and used for reporter gene assay (*right*). Means of three independent experiments performed at least in triplicate are shown. **P < 0.01; ***P < 0.001. **C.** The N2IC-expressing SC-M1/myc-N2IC-His cells and SC-M1/pcDNA3 control cells were infected with adenoviruses expressing miR-23b or GFP for 48 hours (*left*). SC-M1 cells were infected with adenoviruses expressing miR-23b or GFP for 24 hours after transfection with Ets1-expressing construct pCI-Ets1 or control vector pcDNA3-HA2 for 24 hours (*right*). Then the treated cells were seeded for the assays of tumorsphere formation, colony formation, migration, and invasion as described in the legends to Figure 3 and Figure 4. *P < 0.05; **P < 0.01; ***P < 0.001. #*P* < 0.05; ##*P* < 0.01. **D.** Notch2 receptor-knocked down SC-M1/Notch2i cells (#1 and #9) and SC-M1/Luci control cells were transfected with antagomir-23b (anti-miR-23b) or scrambled control (−) for 48 hours (*left*). SC-M1 cells were co-transfected with antagomir-23b and siRNA vectors against Ets1 (#917 and #918) for 48 hours (*right*). Then the treated cells were seeded for the assays of tumorsphere formation, colony formation, migration, and invasion. *P < 0.05; **P < 0.01; ***P < 0.001. #*P* < 0.05; ##*P* < 0.01; ###*P* < 0.001. Data are shown as mean ± standard deviation.

To identify miR-23b targets, *in silico* analyses were employed to search for mRNA 3′-UTRs containing the putative miR-23b-binding sites. Using widely used software including DIANAmT, miRDB, PicTar, RNAhybrid, and TargetScan6.2 algorithms, there are conserved putative miR-23b-binding sites in Notch2 receptor (Supplementary Figure S5, *left*) and Ets1 (Supplementary Figure S5, *right*) 3′-UTRs. Protein levels of N2IC and Ets1 were decreased by miR-23b overexpression in SC-M1, AZ521, and NUGC-3 cells (Figure 5A).

To further check whether miR-23b targets Notch2 receptor and Ets1 3′-UTRs, reporter gene assay was performed. After transfection with the reporter plasmids containing human Notch2 receptor or Ets1 3′-UTRs, reporter gene activities were attenuated by miR-23b overexpression in SC-M1 cells (Figure 5B). These data clearly demonstrate that miR-23b regulates Notch2 receptor and Ets1 expressions through targeting their 3′-UTRs.

It was found that Notch2 pathway [4] and Ets1 [21] contribute to gastric cancer progression and metastasis. To delineate whether the miR- 23b-suppressed progression of gastric cancer cells is through down-regulating Notch2 receptor or Ets1, the miR-23b-insensitive N2IC-expressing SC-M1/myc-N2IC-His cells and SC-M1 cells transfected with miR- 23b-insensitive Ets1-expressing construct were infected with miR-23b-expressing adenoviruses for the subsequent tumorsphere formation, colony formation, migration, and invasion assays. N2IC and Ets1 overexpressions elevated progression abilities of SC-M1/myc-N2IC-His cells and SC-M1 cells, respectively (Figure 5C). The miR- 23b-repressed progression abilities in the treated cells were relieved by N2IC or Ets1 overexpressions. Alternatively, Notch2 receptor knockdown in SC-M1/Notch2i cells or Ets1 knockdown in SC-M1 cells transfected with siRNA vectors against Ets1 impaired progression abilities (Figure 5D). The miR-23b knockdown-mediated increment of progression abilities in the treated cells was abolished after Notch2 receptor or Ets1 knockdowns.

### miR-23b affects pluripotency gene expression and ultra-structure of tumorspheres in SC-M1 cells through Notch2 pathway or Ets1

**Figure 6:**
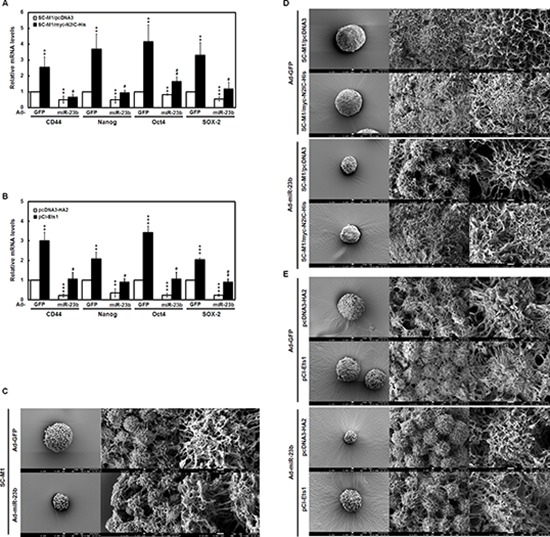
miR-23b affects pluripotency gene expressions and tumorsphere ultra-structure in SC-M1 cells through Notch2 pathway or Ets1 **A-B.** SC-M1/myc-N2IC-His cells or SC-M1/pcDNA3 control cells A and SC-M1 cells transfected with Ets1-expressing construct pCI-Ets1 or control vector pcDNA3-HA2 B were infected with adenoviruses expressing miR-23b (Ad-miR-23b). Then the treated cells were seeded for tumorsphere formation assay as described in the legend to Figure 5D. The transcript levels of CD44, Nanog, Oct4, and SOX-2 in tumorspheres were measured by quantitative real-time PCR and then normalized to GAPDH. **P < 0.01; ***P < 0.001. #*P* < 0.05; ##*P* < 0.01. Data are shown as mean ± standard deviation. **C-E.** SC-M1 cells C SC-M1/myc-N2IC-His cells or SC-M1/pcDNA3 control cells D and SC-M1 cells transfected with Ets1-expressing construct pCI-Ets1 or control vector pcDNA3-HA2 E were infected with adenoviruses expressing miR-23b or GFP for the subsequent tumorsphere formation assay. The ultra-structure of tumorspheres was visualized by scanning electron microscope. Images at 300× (*left*), 3,000× (*middle*), and 10,000× (*right*) magnification are from a representative experiment. Scale bar: left, 10 μm; middle, 1 μm; right, 1 μm.

To further ascertain whether miR-23b modulates the levels of pluripotency genes in tumorspheres of SC-M1 cells through targeting Notch2 receptor or Ets1, quantitative real-time PCR analysis of CD44, Nanog, Oct4, and SOX-2 mRNAs was performed. Data showed that the mRNA levels were elevated by N2IC in tumorspheres of SC-M1/myc-N2IC-His cells (Figure 6A) and by Ets1 in those of SC-M1 cells transfected with Ets1-expressing construct (Figure 6B), but down-regulated by miR-23b in those of control cells. The miR- 23b-suppressed mRNA levels of these pluripotency genes were mitigated by N2IC and Ets1 overexpressions in tumorspheres of SC-M1/myc-N2IC-His cells (Figure 6A) and SC-M1 cells (Figure 6B), respectively.

There is a microvilli-like structure in tumorsphere surface of SC-M1 cells [31]. The effect of miR-23b on tumorsphere ultra-structure in SC-M1 cells was also examined by scanning electron microscope. Results showed that miR-23b overexpression reduced the microvillus extension in tumorsphere surface of SC-M1 cells and affected the tumorsphere ultra-structure (Figure 6C). N2IC and Ets1 overexpressions increased the microvillus extension in SC-M1/myc-N2IC-His cells (Figure 6D) and SC-M1 cells (Figure 6E), respectively. The miR-23b-mediated reduction of microvillus extension and induction of morphological change in tumorspheres were partially rescued by N2IC and Ets1 overexpressions in SC-M1/myc-N2IC-His cells (Figure 6D) and SC-M1 cells (Figure 6E), respectively.

### miR-23b diminishes tumor growth and lung metastasis of SC-M1 cells via Notch2 pathway

**Figure 7:**
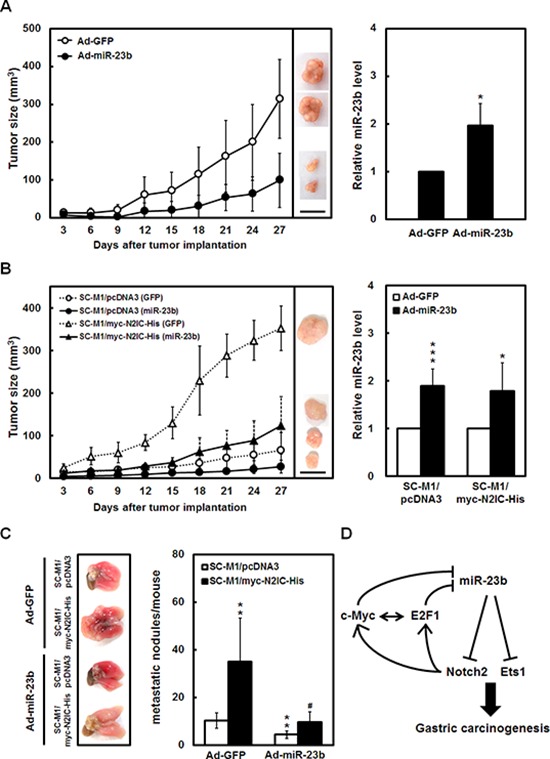
miR-23b diminishes tumor growth and lung metastasis of SC-M1 gastric cancer cells via Notch2 pathway **A-B.** After infection with adenoviruses expressing miR-23b (Ad-miR-23b) or GFP (Ad-GFP) into SC-M1 cells A and SC-M1/myc-N2IC-His cells as well as SC-M1/pcDNA3 control cells B for 48 hours, the viable infected cells were subcutaneously injected into nude mice (*n* = 5 per group) for the measurement of tumor sizes at the time indicated (*left*). On day 27, the mice were sacrificed and subcutaneous tumors were excised for the detection of miR-23b expression using miRNA quantitative real-time PCR (*right*). Data are representative of 3 experiments. Bar, 1.0 cm. *P < 0.05; ***P < 0.001. **C.** For measurement of metastatic nodules in lungs, NOD-SCID mice (*n* = 6 per group) were injected with the viable SC-M1/myc-N2IC-His cells and SC-M1/pcDNA3 control cells by tail vein injection after infection with adenoviruses expressing miR-23b or GFP. After 15 weeks, the mice were sacrificed and the metastatic nodules in the lungs were counted by gross and microscopic examination. Data are from a representative experiment that was performed three times with identical results. **P < 0.01; #*P* < 0.05. Data are shown as mean ± standard deviation. **D.** A model depicting reciprocal regulation between Notch2 receptor and miR-23b in gastric carcinogenesis.

To validate the effect of miR-23b on tumor growth of gastric cancer cells, SC-M1 cells infected with miR-23b-expressing adenoviruses were subcutaneously injected into nude mice to assess the xenografted tumor growth. The tumor sizes of SC-M1 cells were reduced by miR-23b overexpression (Figure 7A, *left*). On day 27 post-injection, subcutaneous tumors were excised from the sacrificed mice for the detection of miR-23b expression by miRNA quantitative real-time PCR. Results of miRNA quantitative real-time PCR showed that miR-23b levels in the xenografted tumors of mice injected with miR-23b-expressing SC-M1 cells were higher than those of control cells (Figure 7A, *right*). Moreover, we checked whether miR-23b-mediated inhibition of tumor growth in SC-M1 cells depends on Notch2 pathway. Data showed that the miR-23b-inhibited xenografted tumor growth was restored in N2IC-expressing SC-M1/myc-N2IC-His cells (Figure 7B, *left*). miR-23b levels in the xenografted tumors of mice injected with miR-23b-expressing cells were higher than those of control cells by quantitative real-time PCR (Figure 7B, *right*).

To evaluate the effect of miR-23b/Notch2 pathway on metastatic colonization of gastric cancer cells, N2IC-expressing SC-M1/myc-N2IC-His cells or control cells were infected with miR-23b-expressing adenoviruses and then intravenously injected into lateral tail vein of non-obese diabetic severe-combined immunodeficiency (NOD-SCID) mice. The injected mice were sacrificed for detection of metastatic nodules in lungs fifteen weeks later. Results showed that mice injected with N2IC-expressing SC-M1/myc-N2IC-His cells had numerous large metastases in lungs as compared with those injected with control cells (Figure 7C). miR-23b overexpression decreased the number of metastases in lungs of mice injected with control cells. The miR-23b-reduced ability to form metastases in lungs was alleviated by N2IC overexpression.

## DISCUSSION

To decipher the molecular mechanism regarding the regulation of gastric cancer progression, we had identified the Notch2 receptor-related miRNA miR-23b and further characterized its role in gastric carcinogenesis herein. The critical epigenetic regulator miR-23b exhibits a tumor-suppressive effect on gastric cancer progression through targeting Notch2 receptor and Ets1. Additionally, Notch2 pathway and miR-23b form a reciprocal regulation loop in gastric carcinogenesis (Figure 7D). The mutual regulation of Notch2 pathway and miR-23b in this loop could more precisely tune target gene expressions.

miR-23b exhibits dichotomous roles in cancer progression [32]. For example, miR-23b promotes tumorigenesis of breast cancer cells [32, 33]. Conversely, miR-23b inhibits carcinogenesis in prostate cancer cells [34], colon cancer [35], and hepatocellular carcinoma [36] and suppresses lymph node metastasis in breast cancer [37]. We clearly demonstrated herein that miR-23b blocks gastric cancer progression. Additionally, numerous reports documented that miRNAs regulate the stemness of cancer stem cells [18, 31, 38]. Notch2 pathway and miR-205 form a mutual regulation loop in modulating mammary stem cell fate [18]. Results of this study also showed that miR-23b dampened tumorsphere formation ability (Figure 5) and affected pluripotency gene expressions and tumorsphere ultra-structure (Figure 6) in gastric cancer cells through Notch2 receptor. These results imply that the Notch2/miR-23b reciprocal loop plays an essential role in the maintenance of cancer stem-like phenotype in gastric cancer cells.

The role of E2F1 in tumorigenesis is complicated [19] and their expressions are higher in samples of gastric cancer patients with stages I–II but lower in those with stages III–IV [20]. Based on our data of stratified analysis (Figure 1B), mRNA levels of E2F1 were also down-regulated in gastric cancer samples of patients with advanced stages. Furthermore, there are several approaches regarding preclinical evaluation and clinical trials of strategies for Notch pathway modulation [3]. To develop miRNA-based therapy for cancer treatment, it is foreseeable to suppress miRNAs acting as oncogenes or restore those acting as tumor suppressors in the future [8]. Based on the results of Figure 1C, miR-23b has the potential as the diagnostic marker and therapeutic target of gastric cancer with lymph node metastasis. All E2F1 [19], Notch pathways [2, 3], and miRNAs [8, 9] exert the context-dependent, dichotomous, and contradictory roles in cancers. It is difficult to treat gastric malignancies only *via* cutting-off one factor in Notch2/miR-23b loop. For the treatment of gastric cancer, it is a potential application in combination therapies targeting miR-23b, E2F1, Notch2 receptor, and Ets1 in the future.

## MATERIALS AND METHODS

### Plasmids and plasmid construction

Expression construct pcDNA-myc-N2IC-His contains the cDNA encoding amino acid residues 1700–2471 of human Notch2 receptor [4]. Plasmid pCI-ETS1 contains the cDNA encoding human Ets1 [39]. The DNA segment of human Notch2 receptor 3′-UTR (nucleotide 2365–2932 from the start of 3′-UTR) was amplified by PCR from the genomic DNA of SC-M1 cells to construct reporter plasmid pNotch2 3′-UTR-Luc as described previously [40]. Reporter plasmids of pmiR-RB-Ets1 3′-UTR [21] and miR-23b-27b-24-1 promoter [41] contain the DNA segments of human Ets1 3′-UTR (nucleotide 1–3519 from the start of 3′-UTR) and human miR-23b-27b-24-1 cluster promoter, respectively. Reporter plasmids Nanog-Luc, SOX-2-Luc, and Oct4-Luc contain promoters of human pluripotency genes Nanog, SOX-2, and Oct4, respectively [42].

For knockdown of the endogenous Ets1 and E2F1, their target sequences were constructed in siRNA vector pLKO.1 as described previously [5, 43]. To knock down Notch2 receptor, the target sequences were also constructed in siRNA vector pLKO.1 [4]. The siRNA vector pLKO.1-shLuc against luciferase was used as a negative control for knockdown validation [5, 43]. To construct miR-23b-expressing adenoviral plasmid, its precursor sequence was amplified by PCR from the genomic DNA of SC-M1 cells. Then the precursor DNA fragment was cloned to generate a recombinant miR- 23b-expressing adenoviral plasmid containing a GFP tracer as described [40].

To construct the miR-23b-expressing adenoviral plasmid, the precursor sequence of miR-23b was amplified by PCR from the genomic DNA of SC-M1 cells. The amplified DNA fragment was subsequently cloned into pAdTrack-CMV vector containing a GFP tracer to generate pAdTrack-miR-23b plasmid [40]. After linearizing with Pme I, pAdTrack-miR-23b plasmid was transformed into BJ5183-AD-1 bacterial cells harboring the adenoviral pAdEasy-1 vector (Stratagene) to construct a recombinant miR-23b-expressing adenoviral plasmid. The used primers for plasmid construction, siRNA, PCR, and real-time PCR were listed in Supplementary Tables S1. All constructs used in this study were verified by sequencing.

### Cell culture and transfection

Human stomach carcinoma including SC-M1, AGS, AZ521, NUGC-3, KATO III, SNU-16, and NCI-N87 cells were cultured in RPMI 1640 medium with 10% fetal bovine serum. The stable N2IC-expressing SC-M1/myc-N2IC-His cells, Notch2 receptor-knocked down SC-M1/Notch2i (#1 and #9) cells, and their control cells (SC-M1/pcDNA3 and SC-M1/Luci cells, respectively) were described previously [4]. Cells were transiently transfected by electroporation or transfection reagents such as Lipofectamine™ 2000 (Invitrogen). SC-M1 cells (5 × 10^5^) were seeded onto 6-well plates and then transiently transfected for luciferase reporter gene assay [40]. The transfected cells were infected with adenoviruses expressing miRNAs or GFP after 24 hours post-transfection. Luciferase activity was measured and then normalized after 24 hours post-infection as described before [40]. Antagomir-23b and scrambled control oligonucleotides (Ambion) were transfected into SC-M1 cells at a final concentration of 50 or 100 nM [40].

### Recombinant adenoviruses

The recombinant adenoviral plasmid expressing miR-23b was linearized by Pac I and then transfected into AD-293 adenovirus packaging cells to obtain packaged recombinant adenovirus expressing miR-23b as described before [40]. The pAdTrack-CMV vector was also used to generate GFP-expressing adenoviruses as a control. Both titer and multiplicity of infection of recombinant adenoviruses were detected according to the manufacturer's instructions (Stratagene).

### Quantitative real-time PCR analysis

Total RNA isolated by Trizol reagent (Invitrogen) was used to synthesize cDNA for the detection of mRNAs and then the cDNAs were amplified [31]. All primers used in real-time PCR analysis are listed in the Supplementary Tables S1. The relative quantification of mRNA levels was normalized with those of GAPDH and then corrected to a calibrator using the StepOne software 2.1 (Applied Biosystems). The cDNA synthesis and TaqMan miRNA real-time PCR assays were performed for the detection of mature miRNAs [40]. The relative quantification of miRNA levels was normalized to those of RNU48 small nucleolar RNA and corrected to a calibrator using the StepOne software 2.1.

### Western blot analysis

As described previously [44], whole-cell extracts were prepared for Western blotting using anti-Notch2 C-terminal, anti-E2F1, anti-c-Myc, anti-E-cadherin, anti-Ets1, anti-plakoglobin (Santa Cruz), anti-vimentin (Sigma), anti-N-cadherin (BD Biosciences), anti-CD44, anti-Nanog, anti-Oct4, anti-SOX2, anti-Twist, and anti-GAPDH (GeneTex) antibodies.

### Cell growth and viability assays

To evaluate cell growth, the treated cells (1 × 10^4^) were seeded and then counted by trypan blue exclusion method at the time indicated. Cell viability was assessed by MTT (Sigma-Aldrich) assay after incubation for 24 or 48 hours as described [4].

### Colony and tumorsphere formation assays

For the colony formation assay, the treated cells were seeded for the assay of anchorage-independent growth in soft agar [45]. Two weeks later, the colonies larger than 0.1 mm in diameter were counted from 10 random fields under the microscope. For the tumorsphere formation assay, a total of 300 treated cells were suspended in stem cell-selective conditions and then seeded onto 96-well ultra-low attachment plates [40]. Nine days later, the spheres larger than 50 μm in diameter were counted under the microscope.

### Migration and invasion assays

As described [5], migration and invasion abilities of the treated cells were examined in 24-well plates by Millicell tissue culture plate well inserts (Millipore) for 12 hours and BD BioCoat Matrigel Invasion Chambers (Becton Dickinson) for 20 hours, respectively. After removing cells on the upper surface of the membrane with a cotton swab, the migrated or invaded cells on the lower surface of membrane were fixed with methanol and stained with 0.005% crystal violet in PBS for 1 hour. Then numbers of the migrated or invaded cells were counted from 10 random fields under the microscope.

### Scanning electron microscopic analysis

After washing by PBS twice, the tumorspheres collected by sedimentation were fixed with 2% glutaraldehyde for 2 hours, followed by 1% osmium tetraoxide for 2 hours for ultra-structural analysis [31]. After washing with deionized water, samples were frozen at −80°C for 8 hours and then dehydrated overnight using Freeze-Drying machine (VirTis Freezemobile 25ES). Subsequently, the lyophilized samples were subjected to sputter coating with gold nano particles for 120 seconds before the examination with a scanning electron microscope (JSM-7600F; JEOL Ltd., Japan) at an accelerating voltage of 5 kV.

### Xenografted tumorigenicity assay in nude mice

All animal experiments performed in this study were carried out in accordance with a protocol approved by the institutional ethical committee (Institutional Animal Care and Use Committee of National Yang-Ming University). Five-week-old BALB/c nu/nu mice purchased from the National Science Council Animal Center (Taipei, Taiwan) were subcutaneously injected at both hind limbs with 3 × 10^6^ viable cells in a total volume of 0.1 ml of PBS. Volume of xenografts was estimated after measurement with calipers every 3 days [40]. The injected mice were sacrificed on day 27, and miR-23b levels in excised tumor samples were detected by miRNA quantitative real-time PCR subsequently.

### *In vivo* tail vein metastasis assay

In brief, six-week-old male NOD-SCID mice (National Taiwan University, Taipei, Taiwan) were injected with 1 × 10^6^ viable cells by tail vein injection [40]. After 15 weeks, the injected mice were sacrificed, and the metastatic nodules in lungs of mice were counted by gross and microscopic examination.

### Surgical samples

Gastric adenocarcinoma tissues were collected from gastric cancer patients who underwent gastric resection at the Department of Surgery, Taipei Veterans General Hospital. Among these patients, none had undergone chemotherapy or radiotherapy prior to surgery. Before study, informed consent was obtained from all patients and the analysis of human tissue specimen was approved by the Institutional Review Board in Taipei Veterans General Hospital.

### Statistical analysis

Data analysis was performed using Student's *t*-test for simple comparison of two values. The correlation among of miR-23b, mRNAs, and proteins levels was analyzed using the Pearson correlation analysis. The difference of results was considered statistically significant when the *P* value was less than 0.05.

## SUPPLEMENTARY FIGURES AND TABLE



## Abbreviations

EMT: epithelial-mesenchymal transition
Ets1: v-ets erythroblastosis virus E26 oncogene homolog 1
miR-23b: microRNA-23b
MTT: 3-(4, 5-dimethyl-2-thiazolyl)-2, 5-diphenyl tetrazolium bromide
N2IC: Notch2 receptor intracellular domain
3′-UTR: 3′-untranslated region
TCGA: The Cancer Genome Atlas
