# Supplementary material for: The reciprocal regulation loop of Notch2 pathway and miR-23b in controlling gastric carcinogenesis
Source: Oncotarget. 2015 May 18;6(20):18012–26. doi: 10.18632/oncotarget.4000 (PMC4627232; doi:10.18632/oncotarget.4000)
Supplement: Supplementary file 1 [file oncotarget-06-18012-s001.pdf]

SUPPLEMENTARY FIGURES AND TABLE

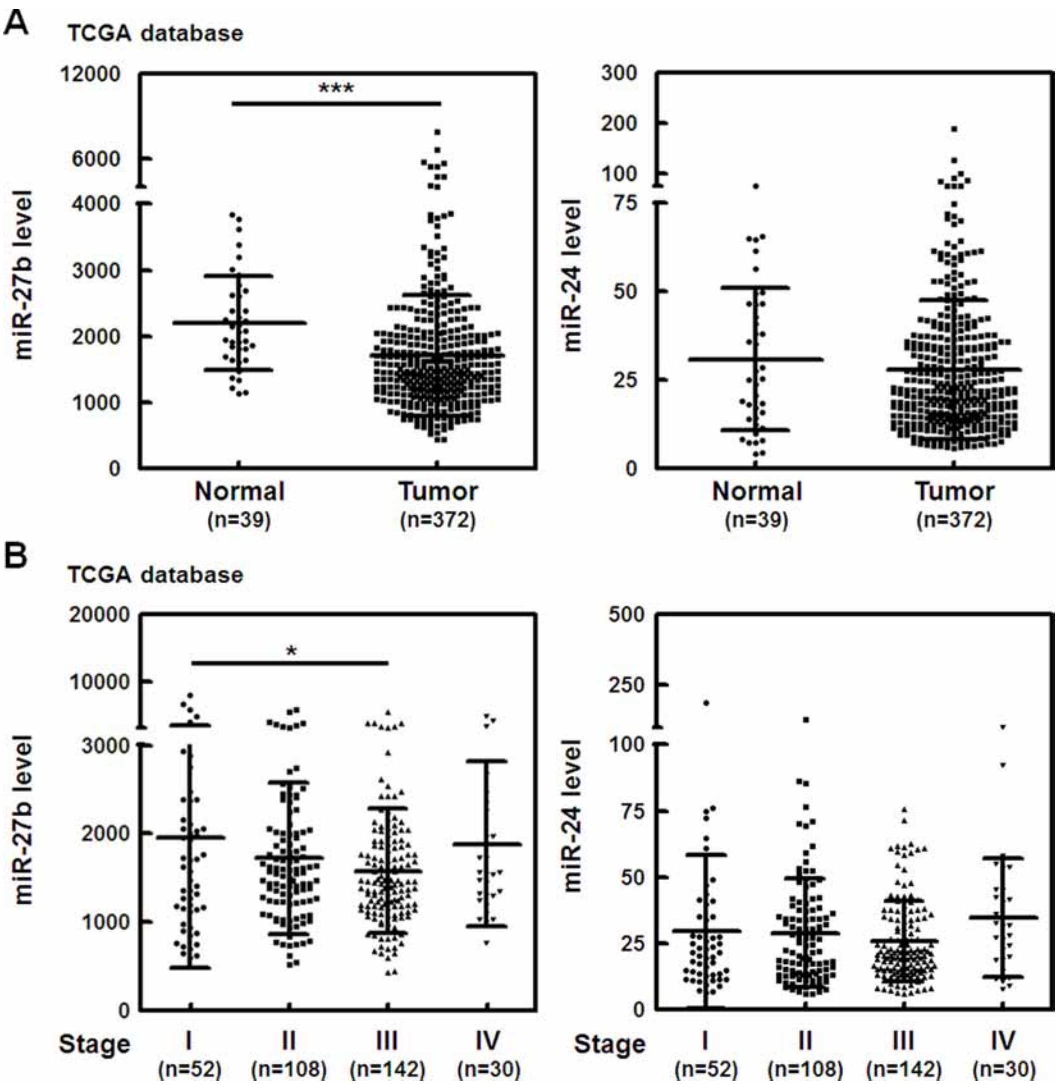

Supplementary Figure S1: Levels of miR-27b but not miR-24 are down-regulated in stomach adenocarcinoma samples. **A.** As described in the legend to Figure 1, level 3 data of miR-27b (*left*) and miR-24 (*right*) expressions from stomach adenocarcinoma samples (n = 372) and normal tissue samples (n = 39) were downloaded from the TCGA and Broad GDAC Firehose data portal and then analyzed for comparing abundances by GraphPad Prism 5 software. ***P < 0.001. **B.** As described above, miR-27b (*left*) and miR-24 (*right*) levels in stomach adenocarcinoma samples were downloaded and then divided according to the stage classification. *P < 0.05. Data are shown as mean ± standard deviation.

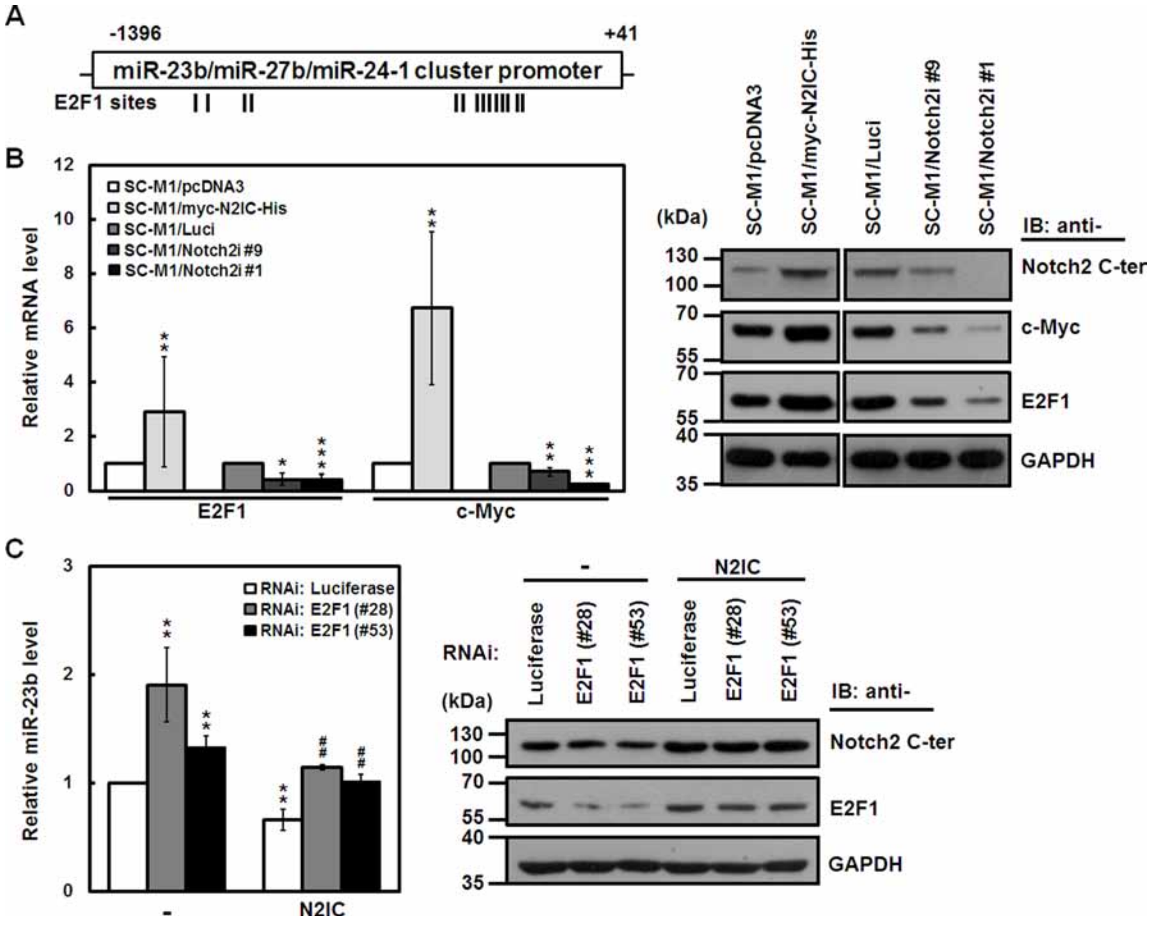

Supplementary Figure S2: N2IC down-regulates miR-23b through inducing E2F1. A. Schematic representation of human miR-23b-27b-24-1 promoter. Black lines represent the 14 putative E2F1-binding sites in this promoter. **B.** The transcript levels of E2F1 and c-Myc in SC-M1/myc-N2IC-His cells, SC-M1/Notch2i cells (#1 and #9), and their control cells (SC-M1/pcDNA3 cells and SC-M1/Luci cells, respectively) were measured by quantitative real-time PCR (*left*). The data were compared, after being normalized to GAPDH. The levels of E2F1 and c-Myc mRNAs in control cells were set to unity. Means of three independent experiments performed at least in triplicate are shown. $*P < 0.05$; $**P < 0.01$; $***P < 0.001$. Whole-cell extracts of these cells were also analyzed by Western blot analysis using anti-Notch2 C-terminal (C-ter), anti-c-Myc, anti-E2F1, and anti-GAPDH antibodies (*right*). **C.** The relative miR-23b levels were determined by miRNA quantitative real-time PCR after co-transfection with N2IC-expressing construct pcDNA-myc-N2IC-His (N2IC) or its control vector pcDNA3.1-myc-His (-) and siRNA vectors against E2F1 (#28 and #53) or luciferase into SC-M1 cells (*left*). The level of miR-23b in SC-M1 cells transfected with siRNA vector against luciferase was set to unity. Means of three independent experiments performed at least in triplicate are shown. $**P < 0.01$. $\#\#P < 0.01$. Data are shown as mean ± standard deviation. Whole-cell extracts of these cells were also analyzed by Western blot analysis using anti-Notch2 C-terminal (C-ter), anti-E2F1, and anti-GAPDH antibodies (*right*).

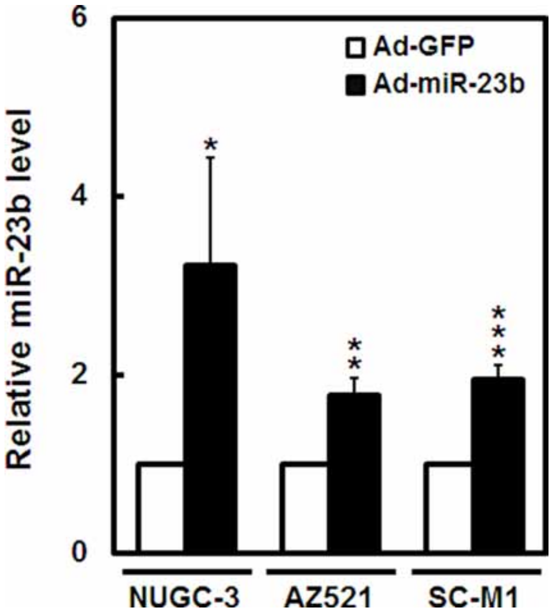

Supplementary Figure S3: Establishment of miR-23b-expressing adenoviral system in NUGC-3, AZ521, and SC-M1 gastric cancer cells. NUGC-3, AZ521, and SC-M1 cells were infected with adenoviruses expressing miR-23b (Ad-miR-23b) or GFP (Ad-GFP) for 48 hours. Then the relative levels of miR-23b were determined using miRNA quantitative real-time PCR. The levels of miR-23b in the cells infected with adenoviruses expressing GFP were set to unity. $^*P < 0.05$; $^{**}P < 0.01$; $^{***}P < 0.001$. Data are shown as mean ± standard deviation.

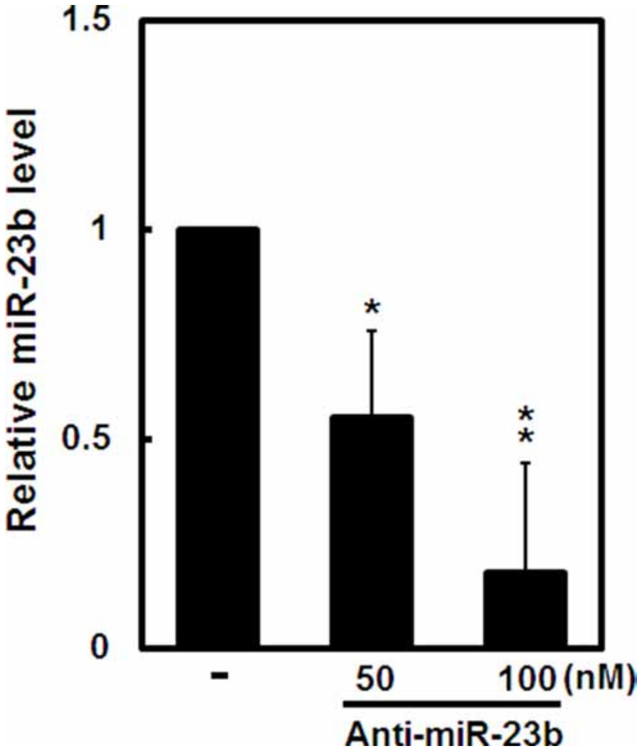

Supplementary Figure S4: Knockdown of miR-23b in SC-M1 cells after transfection with antagomir-23b. SC-M1 cells were transfected with 50 or 100 nM antagomir-23b (anti-miR-23b) or scrambled control (-) for 48 hours. The levels of miR-23b in the transfected cells were measured by miRNA quantitative real-time PCR. *$P < 0.05$; **$P < 0.01$. Data are shown as mean ± standard deviation.

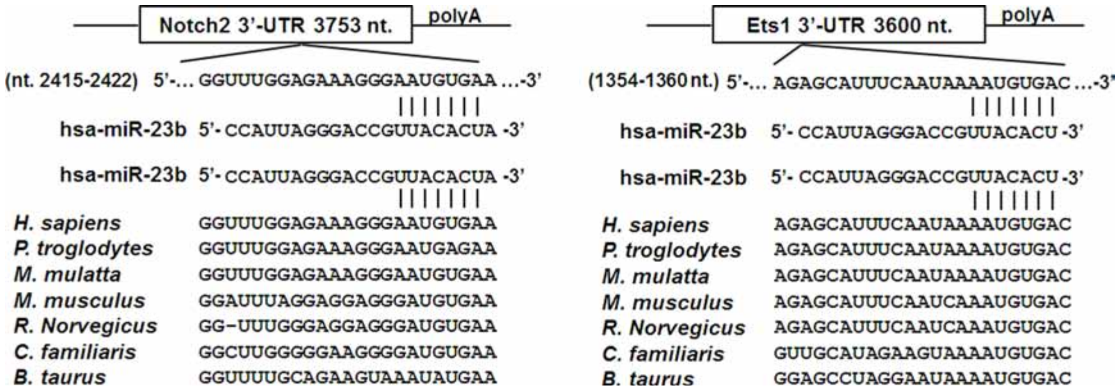

Supplementary Figure S5: The putative miR-23b-binding sites in Notch2 receptor and Ets1 3′-UTRs. There are putative miR-23b-binding sites located at nucleotide 2415 to 2422 from the start of Notch2 receptor 3′-UTR (*left*) and at nucleotide 1354 to 1360 from the start of Ets1 3′-UTR (*right*). The sequences of miR-23b are aligned with the 3′-UTRs of Notch2 receptor and Ets1 in human (*H. sapiens*), chimpanzee (*P. troglodytes*), monkey (*M. mulatta*), mouse (*M. musculus*), rat (*R. norvegicus*), dog (*C. familiaris*), and cow (*B. taurus*).

Supplementary Table S1. Sequence of primers for siRNA, PCR, and real-time PCR

Assays		Sequence (5′ to 3′)	Amplicon (bp)
siRNA	E2F1 (#28)<br>E2F1 (#53)<br>Ets1 (#917)<br>Ets1 (#918)	CTACTCAGCCTGGAGCAAGAA<br>ACCTCTTCGACTGTGACTTTG<br>ATCCCGCTATACCTCGGATTA<br>GACCGTGCTGACCTCAATAAG	
PCR	Notch2 3′-UTR	F TCTAGATCTTTTTCTTGGACTAC<br>R TCTAGATCTCAACAAAACATTAC	566
	Ets1 3′-UTR	F AATTGCTCGAGTGGCACTGAAG<br>R CTAGCGGCCGCCTCTCCAGCAA	3,519
	miR-23b	F ATAAGATCTCCACCTCTTTGCTAG<br>R ATACTCGAGCATCTTCCTCAGCTG	301
real-time PCR	CD44	F TCCAACACCTCCCAGTATGACA<br>R GGCAGGTCTGTGACTGATGTACA	83
	Nanog	F CCTGTGATTTGTGGGCCTG<br>R GACAGTCTCCGTGTGAGGCAT	78
	Oct4	F GGTGGAGGAAGCTGACAACAA<br>R AAATTCTCCAGGTTGCCTCTCA	123
	SOX-2	F GTATCAGGAGTTGTCAAGGCAGAG<br>R TCCTAGTCTTAAAGAGGCAGCAAAC	78
	E2F1	F AGCTGGACCACCTGATGAAT<br>R GTCCTGACACGTCACGTAGG	95
	Ets1	F TCACTAAAGAACAGCAACGA<br>R ATTCACAGCCCACATCAC	92
	Notch2	F GTGAGGGAGACATCAACGAG<br>R GTAAAGGCACTACGGCAAAC	109
	GAPDH	F AAATCCCATCACCATCTTCC<br>R TCACACCCATGACGAACA	194
